# Supplementary material for: Genetically Modified Mesenchymal Stromal/Stem Cells as a Delivery Platform for SE-33, a Cathelicidin LL-37 Analogue: Preclinical Pharmacokinetics and Tissue Distribution in C57BL/6 Mice
Source: Antibiotics (Basel). 2025 Apr 24;14(5):429. doi: 10.3390/antibiotics14050429 (PMC12108424; doi:10.3390/antibiotics14050429)
Supplement: Supplementary file 1 [file antibiotics-14-00429-s001.zip › antibiotics-3553340-supplementary.pdf]

SUPPLEMENTARY MATERIALS

Genetically Modified Mesenchymal Stromal/Stem Cells as a Delivery Platform for SE-33, a Cathelicidin LL-37 Analogue: Preclinical Pharmacokinetics and Tissue Distribution in C57BL/6 Mice

Gasanov V.A.o.[1], Kashirskikh D.A.[1], Khotina V.A.[1], Lee A.A. [1], Nikitochkina S.Y.[1], Kuzmina D.M.[2], Mukhina I.V.[2], Vorotelyak E.A.[1,3], Vasiliev A.V.[1]

[1] Koltzov Institute of Developmental Biology of Russian Academy of Sciences, Moscow, Russia; gasanov-vagif@gmail.com

[2] Department of Normal Physiology, Privolzhsky Research Medical University of Ministry of Health of the Russian Federation, Nizhny Novgorod, Russia; mukhinaiv@mail.ru

[3] Department of Cell Biology, Biological Faculty, Lomonosov Moscow State University, Moscow, Russia; vorotelyak@yandex.ru

Correspondence:

Gasanov Vagif Ali oglu, Khotina Victoria Alexandrovna

Core Centrum «Genomic Technologies Group», Koltzov Institute of Developmental Biology of Russian Academy of Sciences, Moscow, Russia

E-mail: gasanovvagif@gmail.com (G.V.A.o.); v.a.khotina.bio@gmail.com (K.V.A.)

Figure S1. Representative HPLC chromatograms of serum samples from mice following single intravenous administration.

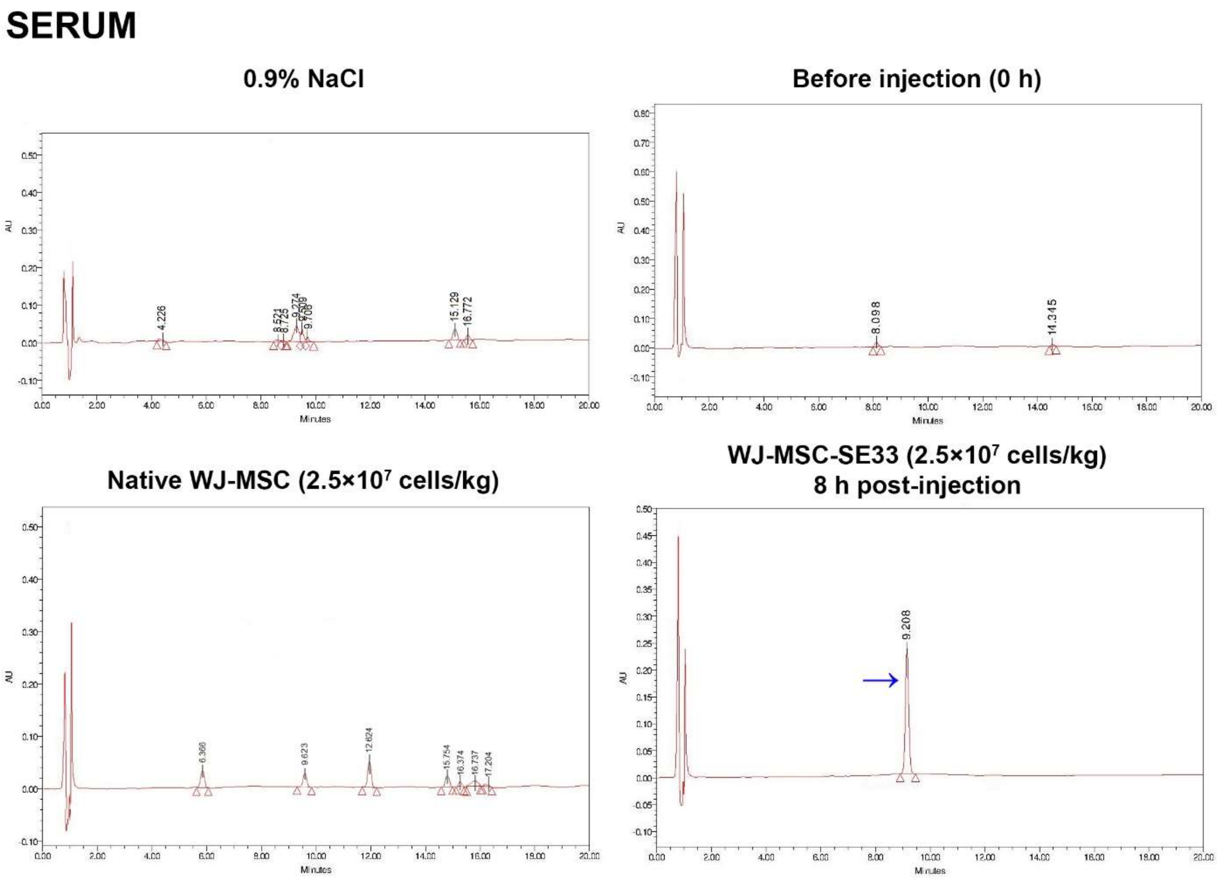

Note: 0.9% NaCl control group; native WJ-MSC control group; Pre-injection baseline (0 h) before WJ-MSC-SE33 administration; 8 hours post WJ-MSC-SE33 administration. The chromatographic peak at 9.10-9.20 min (indicated by blue arrow) corresponds to the SE-33 peptide. This peak is absent in control groups.

Figure S2. Representative HPLC chromatograms of lung samples from mice following single intravenous administration.

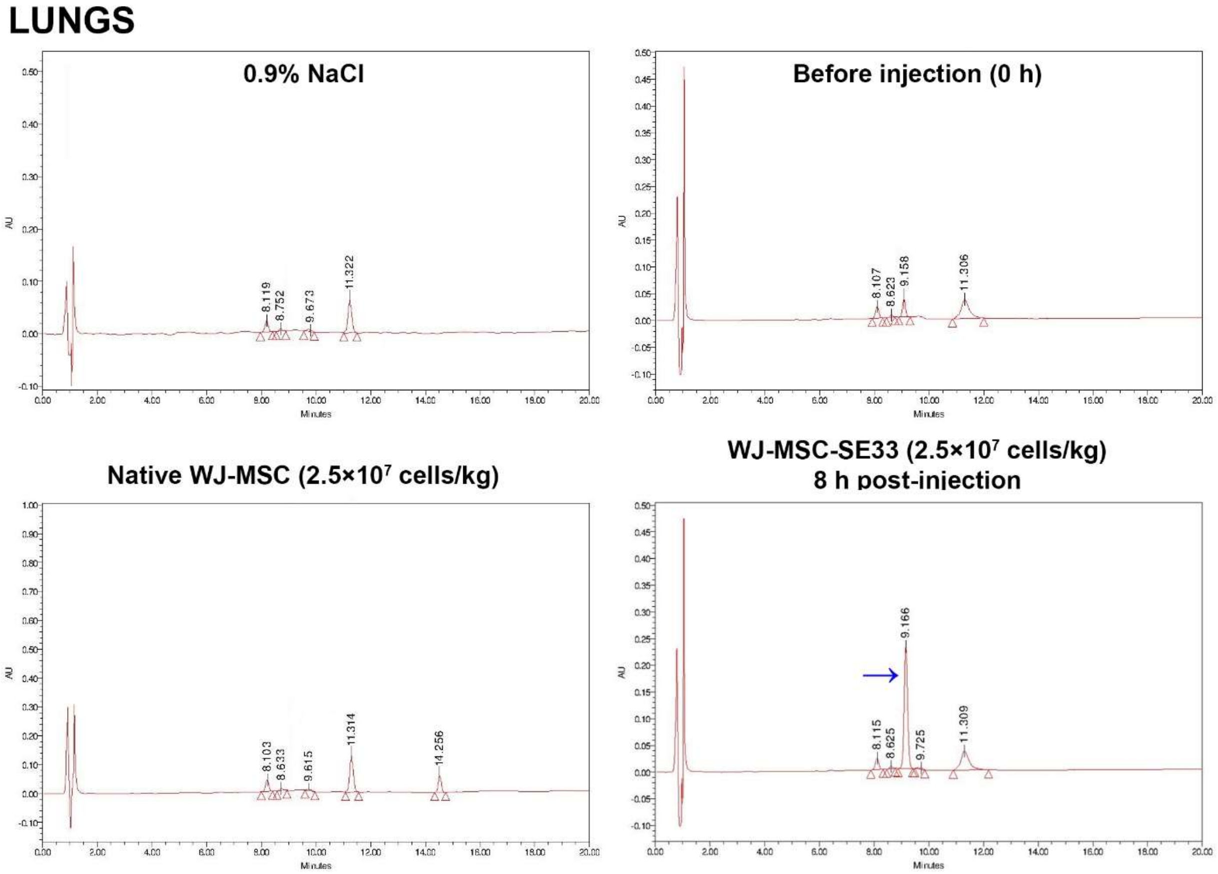

Note: 0.9% NaCl control group; native WJ-MSC control group; Pre-injection baseline (0 h) before WJ-MSC-SE33 administration; 8 hours post WJ-MSC-SE33 administration. The chromatographic peak at 9.10-9.20 min (indicated by blue arrow) corresponds to the SE-33 peptide. This peak is absent in control groups.

Figure S3. Representative HPLC chromatograms of liver samples from mice following single intravenous administration.

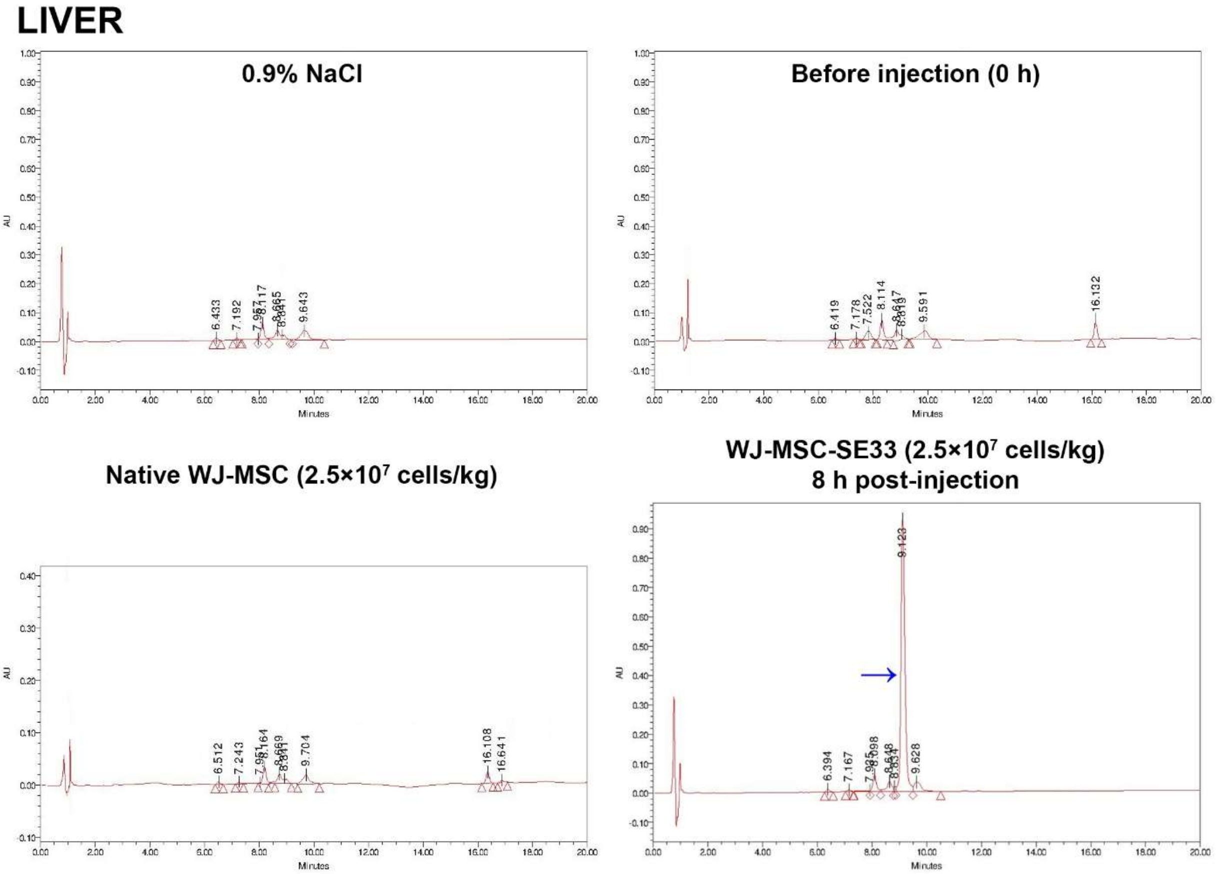

Note: 0.9% NaCl control group; native WJ-MSC control group; Pre-injection baseline (0 h) before WJ-MSC-SE33 administration; 8 hours post WJ-MSC-SE33 administration. The chromatographic peak at 9.10-9.20 min (indicated by blue arrow) corresponds to the SE-33 peptide. This peak is absent in control groups.

Figure S4. Representative HPLC chromatograms of spleen samples from mice following single intravenous administration.

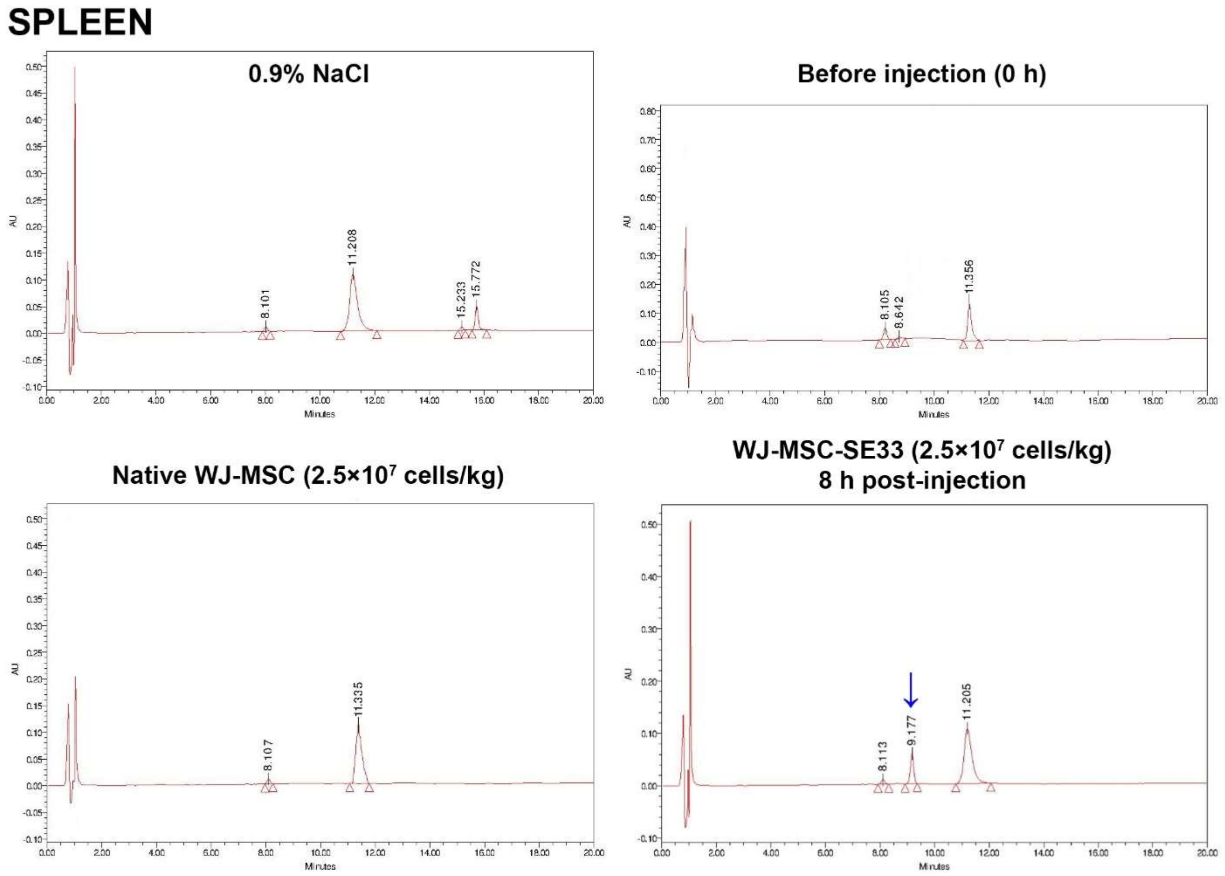

Note: 0.9% NaCl control group; native WJ-MSC control group; Pre-injection baseline (0 h) before WJ-MSC-SE33 administration; 8 hours post WJ-MSC-SE33 administration. The chromatographic peak at 9.10-9.20 min (indicated by blue arrow) corresponds to the SE-33 peptide. This peak is absent in control groups.

Figure S5. Representative HPLC chromatograms of spleen samples from mice following repeated intravenous administrations.

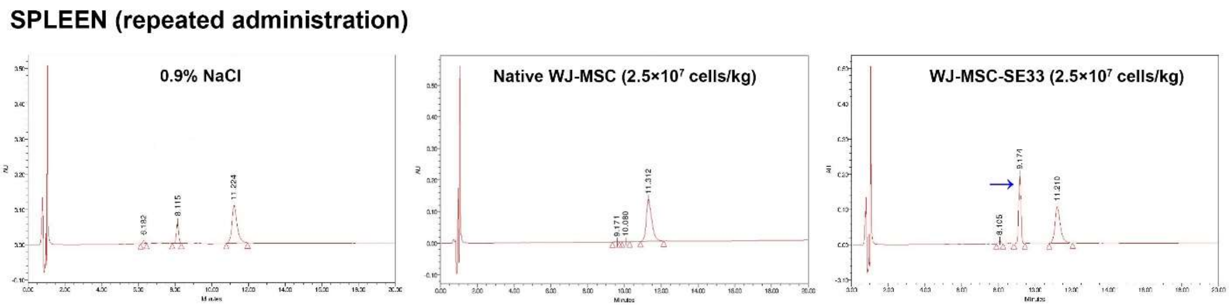

Note: 0.9% NaCl control group; native WJ-MSC control group; Pre-injection baseline (0 h) before WJ-MSC-SE33 administration; 8 hours post WJ-MSC-SE33 administration. The chromatographic peak at 9.10-9.20 min (indicated by blue arrow) corresponds to the SE-33 peptide. This peak is absent in control groups.

Figure S6. Representative HPLC chromatograms of lung samples from mice following single intravenous administration of WJ-MSC-SE33 (preliminary study).

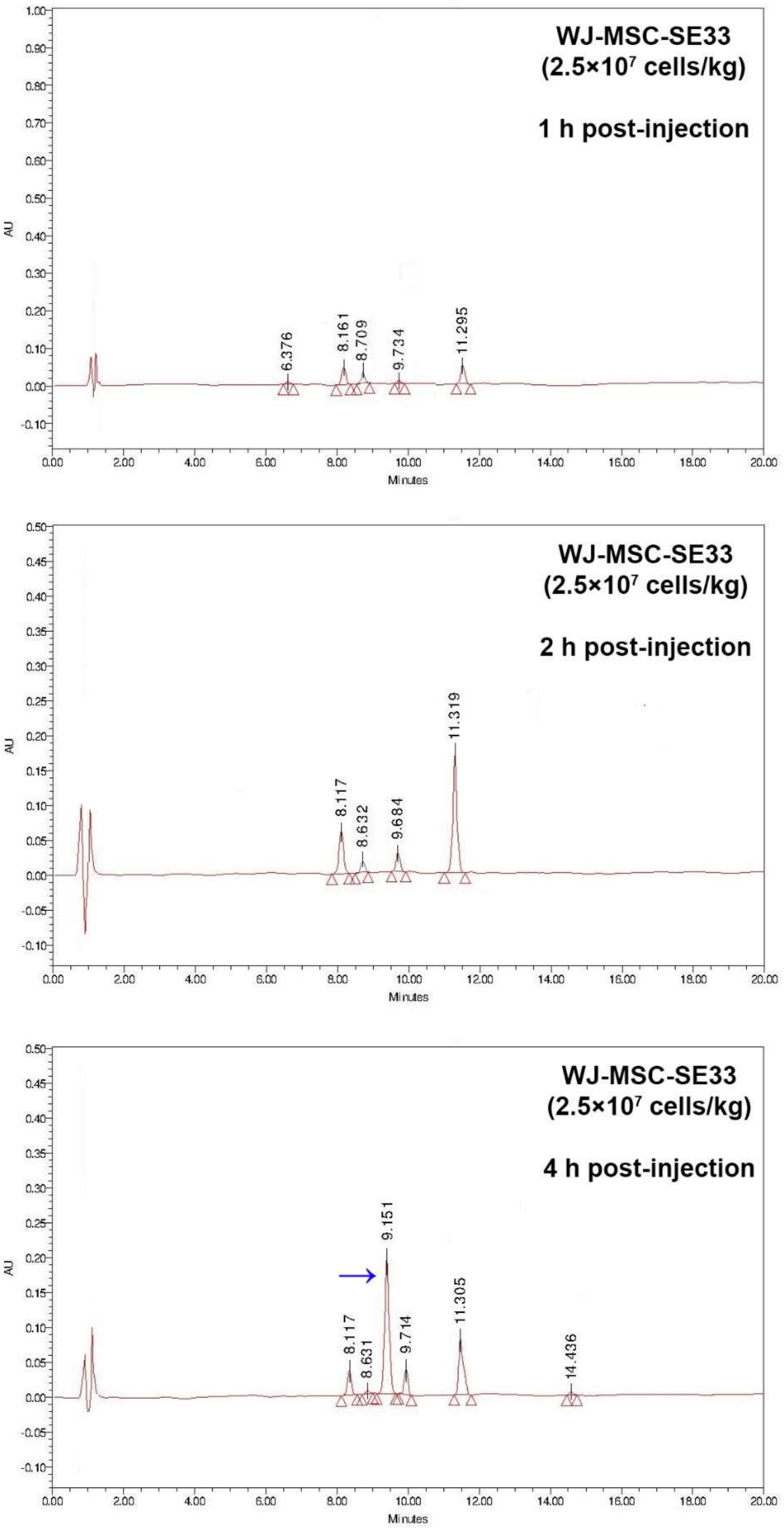

Note: The chromatographic peak at 9.10-9.20 min (indicated by blue arrow) corresponds to the SE-33 peptide. No detectable peak is observed 1 and 2 h post-administration.

Figure S7. Analytical validation of SE-33 peptide detection method.

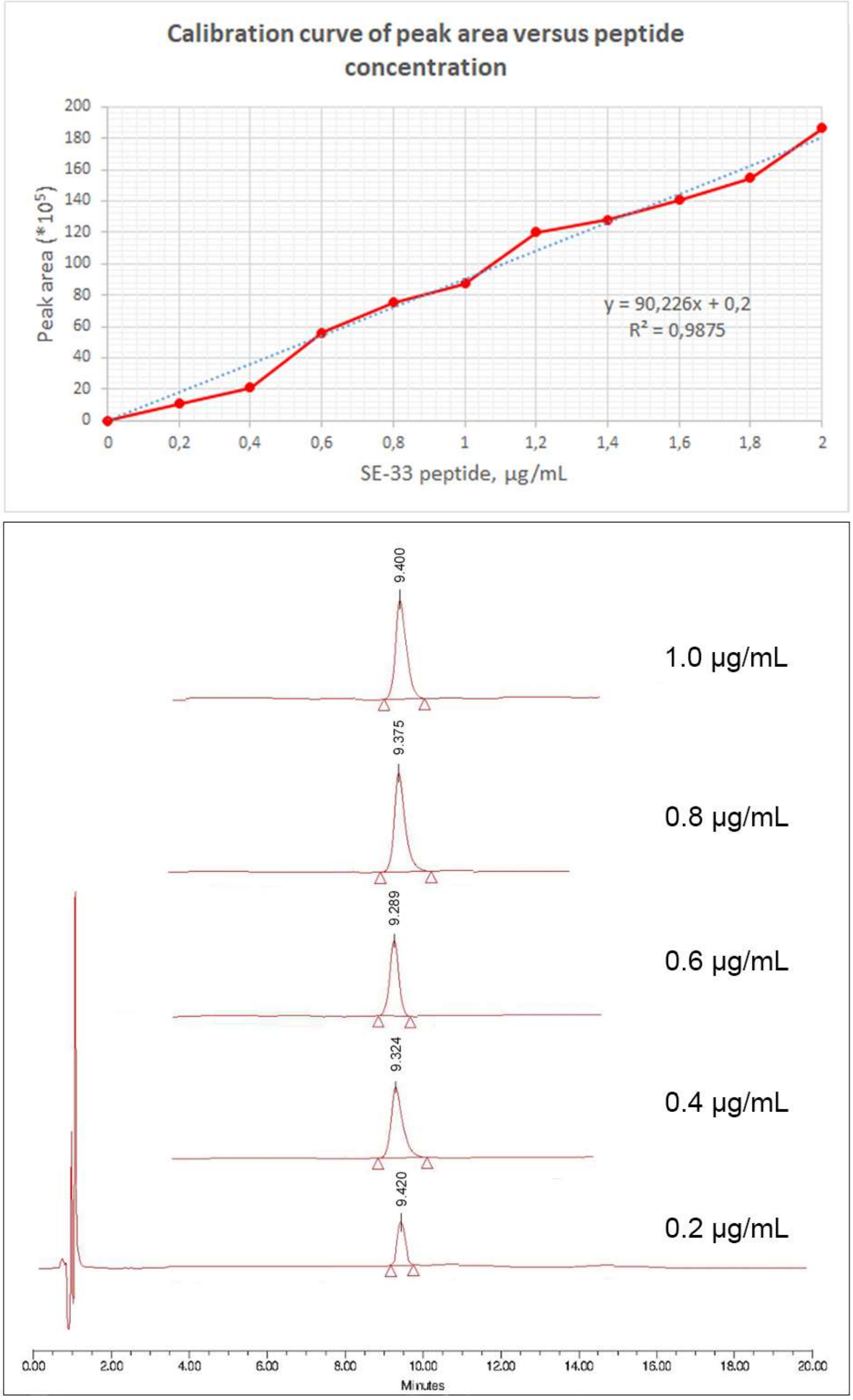

Upper panel: Calibration curve plotting peak area versus SE-33 peptide concentration ($R^2 = 0.9875$). Lower panel: Representative HPLC chromatograms of serially diluted SE-33 (retention time: 9.25-9.50 min).
